# The Role of Midkine in Arteriogenesis, Involving Mechanosensing, Endothelial Cell Proliferation, and Vasodilation

**DOI:** 10.3390/ijms19092559

**Published:** 2018-08-29

**Authors:** Ludwig T. Weckbach, Klaus T. Preissner, Elisabeth Deindl

**Affiliations:** 1 Medizinische Klinik und Poliklinik I, Klinikum der Universität, LMU Munich, 81377 Munich, Germany; Ludwig.Weckbach@med.uni-muenchen.de; 2 Institute of Cardiovascular Physiology and Pathophysiology, Biomedical Center, LMU Munich, 82152 Planegg-Martinsried, Germany; 3 Walter-Brendel-Centre of Experimental Medicine, University Hospital, LMU Munich, 81377 Munich, Germany; 4 Institute of Biochemistry, Medical School, Justus-Liebig-University, 35392 Giessen, Germany; klaus.t.preissner@biochemie.med.uni-giessen.de

**Keywords:** midkine, nitric oxide synthases, VEGF, endothelial-cell proliferation, vasodilation, arteriogenesis, hypoxia, shear stress, reactive oxygen species, tumor

## Abstract

Mechanical forces in blood circulation such as shear stress play a predominant role in many physiological and pathophysiological processes related to vascular responses or vessel remodeling. Arteriogenesis, defined as the growth of pre-existing arterioles into functional collateral arteries compensating for stenosed or occluded arteries, is such a process. Midkine, a pleiotropic protein and growth factor, has originally been identified to orchestrate embryonic development. In the adult organism its expression is restricted to distinct tissues (including tumors), whereby midkine is strongly expressed in inflamed tissue and has been shown to promote inflammation. Recent investigations conferred midkine an important function in vascular remodeling and growth. In this review, we introduce the midkine gene and protein along with its cognate receptors, and highlight its role in inflammation and the vascular system with special emphasis on arteriogenesis, particularly focusing on shear stress-mediated vascular cell proliferation and vasodilatation.

## 1. Introduction

After the discovery of the cytokine midkine (MK) in 1988, its main function was believed to orchestrate embryonic development [1]. However, multiple studies during the last three decades clearly indicated a functional impact of MK on the adult organism as well. By far, most of the studies on MK addressed its role in malignant diseases and suggested a detrimental effect for the host [2,3,4,5]. A significant part of the literature on MK has shown its ability to promote inflammatory responses. Different mechanisms of how MK could reinforce acute or chronic inflammation have been proposed as well and will be partially reviewed here [6]. In addition, recent studies demonstrated that MK serves as a major regulator of angiogenesis and arteriogenesis during pathological conditions in the vascular system. In light of the fast-growing burden of cardiovascular diseases, which are predominantly associated with atherosclerosis and occlusive arterial disease, e.g., in the heart, interventional strategies to modulate arteriogenesis, i.e., the growth of pre-existing arteriolar connections bypassing an occluded artery, may become novel noninvasive treatment modalities for patients with ischemia-related pathologies. In this review, we will summarize the recent findings on the functional impact of MK on the vascular system with a special focus on arteriogenesis and vascular remodeling. 

## 2. Midkine

### 2.1. MK Gene and Protein

In 1988 Kadomatsu and colleagues discovered MK in retinoic acid-differentiated mouse embryonic carcinoma cells, an experimental model to study the early steps of embryogenesis [1]. Expression of MK was upregulated exclusively during the midgestation period in mouse embryogenesis [7]. This fact, together with a predominant expression of MK in the kidneys of adult organisms, finally led to the name mid-kine (mid-gestation, ki-dney) [6,7]. The mouse MK gene is located on chromosome 2, while the human gene, consisting of four coding exons with seven mRNA isoforms [8], was identified on chromosome 11 [9,10]. Among a retinoic acid response element and a binding site for the product of the Wilms tumor-suppressor gene WT-1, a hypoxia-responsive element has been identified in the promoter region of the MK gene [11,12,13].

**Figure 1:**
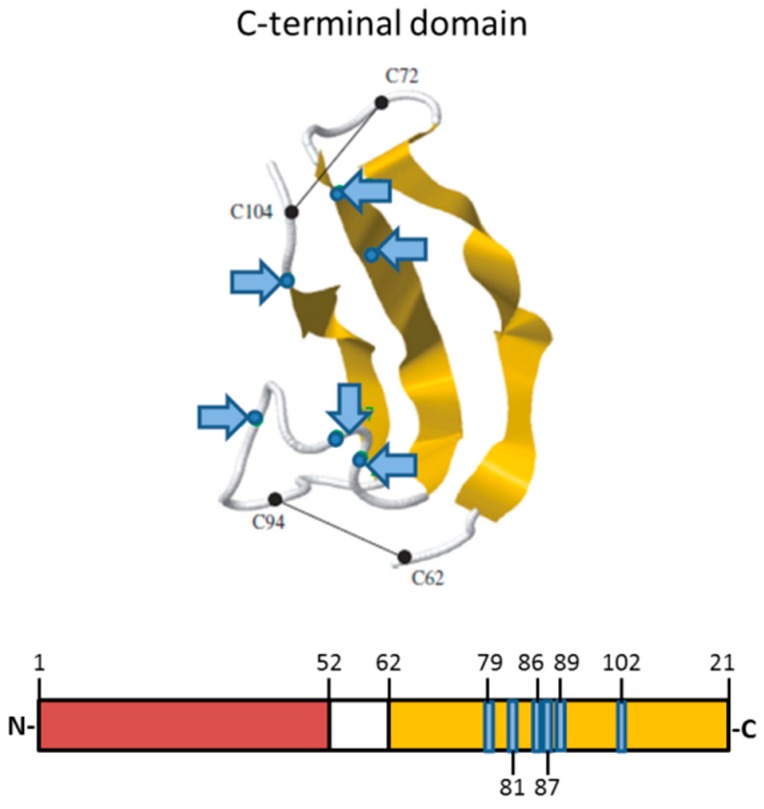
Protein structure of midkine (MK). The protein consists of an N-terminal domain (red) and a C-terminal domain (yellow), which are connected by a hinge region (white). The heparin-binding domains, which consist of basic amino acids, are located in the C-terminal domain (labeled in blue, blue arrows). Adapted from Weckbach et al., 2011 [6], with the permission of the authors.

The human MK protein—composed of 121 amino acids—is a heparin-binding growth factor with a molecular weight of 13 kDa consisting of two similar domains, an N-terminal (amino acids 1–52) and a C-terminal domain (amino acids 62–121), which are connected by a hinge domain (amino acids 53–61) [14]. Each domain contains three antiparallel β-strands [14]. Ten cysteine residues, which are highly conserved among different species, form five disulfide bonds (two bonds in the C-terminal domain and three bonds in the N-terminal domain), building the structure of the domains [14]. MK is a positively charged protein containing two heparin-binding clusters with a number of arginine and lysine residues (cluster 1: K79, R81, K102; cluster 2: K86, K87, R89), located in the C-terminal domain (Figure 1) [6,14]. MK may interact via its positively charged heparin-binding sites with heparin-like glycosaminoglycans of endothelial cells, enabling MK to be immobilized on the luminal site of the vessel wall [15,16].

MK together with pleiotrophin (PTN) forms its own so-called MK family [17], which is structurally not related to any other protein family. Both proteins show a 50% sequence identity and complete conservation of positions of all cysteine residues [18]. The heparin-binding sites are also conserved in MK and PTN with only one basic amino acid exchanged (K84 in MK, R84 in PTN) [19]. A phylogenetic analysis showed a high conservation of the MK protein among different species with an amino acid sequence identity between human and murine MK of 87% [20]. In zebrafish, a very popular and established experimental animal model organism for a variety of human diseases, two MK molecules, midkine-a and midkine-b, have been discovered and most likely represent gene duplicates [21]. *Drosophila melanogaster* expresses two proteins sharing similarities with MK and PTN, named miple-1 and miple-2. Both proteins predominantly resemble the C-terminal domain of MK [22].

### 2.2. MK Receptors

For MK, a wide variety of receptors have been identified (Table 1), which are believed to function as receptor complexes [6]. In general, MK receptors, co-receptors and additional components assemble to form a specific functional receptor complex to promote particular functions of MK. There is evidence that MK signaling via the receptor-like protein tyrosine phosphatase β/protein tyrosine phosphatase ζ (PTPζ) involves phosphatidylinositol 3-kinase (PI3K), *mitogen-activated protein* kinase (MAPK), Src family kinases, and protein kinase C as downstream-signaling partners of MK [23,24]. PI3K and MAPK signal transduction was also observed upon MK binding to anaplastic lymphoma kinase (ALK), suggesting a common signaling pathway for at least some MK receptors [25]. Furthermore, members of the low-density lipoprotein (LDL) family, including the LDL-receptor-related protein-1 (LRP-1), megalin/brushin, LRP-6, or apoE receptor-2 have been shown to act as MK receptors [26]. MK binding to LRP-1 led to a conformational change of β_2_ integrins [27], a prerequisite for neutrophil adhesion during acute inflammation, as detailed below. Although MK did not directly bind to β_1_ integrins on neutrophils [27], MK mediates migration of UMR-106 osteoblast-like cells via α_4_β_1_ integrins and neurite outgrowth of embryonic neurons via α_4_β_1_ integrins, respectively [28]. MK has been shown to bind with high affinity to different glycosaminoglycans including heparan sulfate-trisulfated units and chondroitin sulfate E [29]. In addition, members of the heparan sulfate proteoglycan families of syndecans and glypicans serve as cellular-binding components for MK during neuronal development [30,31,32]. The binding of MK to Notch2 resulted in the upregulation of nuclear factor kappa-light-chain-enhancer of activated B cells (NFκB), implying that MK is involved in inflammatory reactions [33] by, e.g., upregulating the expression of intercellular adhesion molecule-1 (ICAM-1) [34]. Whether the altered combination of components in receptor complexes leads to the modification of MK-dependent signaling in cells remains to be investigated. Moreover, further receptors of MK might be identified in the future.

### 2.3. Sources of MK and Levels of the Protein in the Vascular System

MK is present in the serum and plasma of healthy subjects and levels may become elevated in patients [36]. Yet, the cellular source of MK in the vascular system has not been fully elucidated. Elevated MK serum levels are found in patients with different tumor entities and have been shown to correlate with poor prognosis and recurrence after tumor removal [2,3,4]. The tumor cells may be the source of MK in this case, as malignant tissue often shows high MK expression [5]. The low plasma levels of MK in healthy subjects are rapidly elevated upon administration of heparin. These high post-heparin plasma levels of MK are an indication for the storage of MK in or its association with vascular cells via heparan sulfate proteoglycans [16]. This feature of MK is shared by other heparin-releasable proteins, such as chemokines, antithrombin, or lipoprotein lipase, which bind via their heparin-binding sites to the luminal phase of the endothelium [16]. After administration, heparin in the circulation may compete for these solid-phase heparin-binding sites on the vessel wall and result in protein mobilization. 

Although it is not established which cells provide the source of MK in the vascular system in vivo, endothelial cells are able to synthesize MK under resting conditions in vitro [16]. Moreover, the release of MK into the cell supernatants was elevated under hypoxic conditions in vitro [37]. Immune cells may also represent a vascular source of MK, whereby increased MK expression was detected in isolated human neutrophils and monocytes upon hypoxia. Although earlier studies in the adult murine system suggested a restricted expression of MK to the kidney [7], MK was also detected in the plasma of an anephric patient and elevated in the circulation after heparin administration, indicating that the kidney does not represent the sole source of circulating MK [16]. The exact location and regulation of MK expression in the vascular system still needs to be studied.

### 2.4. MK in Inflammation

Due to the distinct expression pattern of MK during embryonic development, MK was initially believed to predominantly regulate embryogenetic processes. However, the role of MK in situations of acute and chronic inflammation has been increasingly acknowledged.

Under physiological conditions, MK is restrictively expressed in the adult organism. In contrast, inflamed synovial tissue of patients with rheumatoid arthritis showed strong MK expression, whereas no MK expression was detected in healthy control tissue [38]. Elevated MK expression was also observed in kidneys of patients with diabetic nephropathy, a pathological condition that is associated with tubulointerstitial and glomerular inflammation [39]. Inflammation caused by injury after implantation of vascular stents led to increased MK expression in the injured vascular wall compared to control vessels, in which MK could hardly be detected [40]. In a mouse model of experimental autoimmune encephalomyelitis (EAE), which has often been used to study the process underlying multiple sclerosis, MK mRNA was elevated in the spinal cord of EAE mice compared to control mice [41]. These examples demonstrate increased expression of MK under inflammatory conditions in different organs or tissues.

A common feature of inflammatory models using MK-deficient animals was reduced infiltration of leukocytes into the inflamed tissue, which was accompanied by reduced tissue damage [42,43,44]. The underlying cellular and molecular mechanisms of a decreased leukocyte infiltration and subsequent inflammation in the absence of MK were addressed by Weckbach et al. [27]: During the leukocyte recruitment process in postcapillary venules of the inflamed cremaster muscle (a well-established model to study leukocyte trafficking in vivo), the adhesion and subsequent extravasation steps were significantly impaired in MK-deficient mice compared to control animals [27]. In vitro, immobilized MK was able to promote adhesion of isolated neutrophils by stimulating a high affinity state of β_2_ integrins on neutrophils, which is essential for the firm adhesion and transmigration process of these cells [45]. 

While different receptor (complexes) for MK have been identified to be operative in various biological situations [6], the MK-mediated promotion of high-affinity-state β_2_ integrins was found to be abolished by blocking LRP-1 using the receptor-associated protein (RAP), suggesting that the MK-LRP-1 axis was involved in leukocyte adhesion to inflamed endothelium [27]. LRP1 is a large transmembrane receptor consisting of a large extracellular domain (515 kDa), non-covalently linked to a transmembrane domain (85 kDa). Four cysteine-rich complement-like repeats (CR) represent the majority of binding sites for a variety of non-related extracellular ligands, and most of them bind to CRII and CRIV of LRP-1 [46,47]. Based on its interaction with CRII of LRP-1 on rolling neutrophils [48] as well as with heparan proteoglycans on the endothelium, immobilized MK appears to be able to engage β_2_ integrins on neutrophils. In fact, an interaction and modification of β_2_ integrin function by LRP1 has been demonstrated in macrophages [49]. Additional mechanistic relations between MK and leukocyte-mediated inflammation have been proposed. Matsuda et al. showed that MK induced T cell expansion and regulated Th1 cell differentiation in a mouse model of lupus nephritis [50]. In a mouse model of EAE, MK increased inflammation by suppressing the development of tolerogenic dendritic cells, thereby inhibiting the differentiation of regulatory T cells [51]. Whether MK also directly affects T cell trafficking—as shown for neutrophils—has not yet been investigated. In addition to its role in modulating leukocyte recruitment during inflammation, MK has been demonstrated to display strong antibacterial activity, especially against gram-positive bacteria [52]. In septic patients, MK levels were significantly elevated compared to healthy controls, whereby the highest serum values were found in patients with gram-positive bacterial infections [53]. Phylogenetic and structural studies suggested that the peptide regions in MK and PTN for anti-microbial activity are conserved in MK orthologues in the zebrafish as well. The orthologue of MK and PTN in *Drosophila melanogaster* miple-2 also showed strong antibacterial properties, suggesting that the function of MK as intrinsic protective factor may be highly conserved among species [52]. There is growing evidence that MK reinforces the immune defense directly and by engaging and guiding leukocytes. However, there are additional functions of MK in the vascular system, which will be in part reviewed below.

## 3. Arteriogenesis

**Figure 2:**
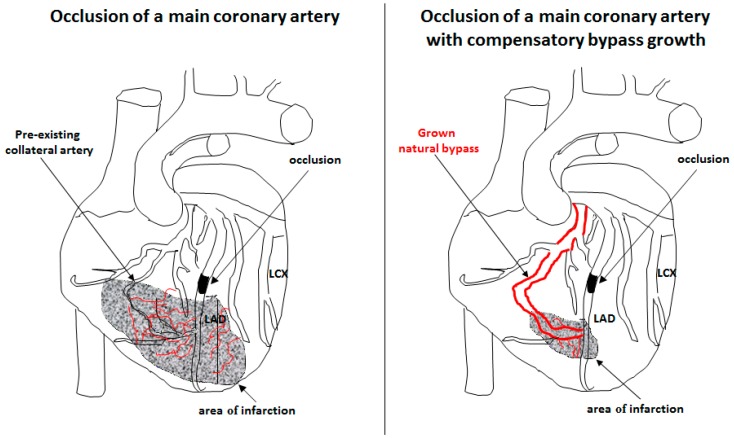
Collateral artery growth. Occlusion of a main coronary artery (**left** picture) results in severe ischemic cardiac-tissue damage (area of infarction) associated with extensive capillary sprouting in order to remove cell debris. If the loss of the occluded artery is compensated by natural bypass growth (arteriogenesis) (**right** picture), the extent of tissue damage and newly formed capillary network is strongly reduced. Coronary arteries: Left Anterior Descending (LAD), Left Circumflex (LCX). Adapted from Deindl et al., 2006 [63], with the permission of *The FASEB Journal*.

Coronary heart disease, stroke, and peripheral artery diseases, commonly referred to as cardiovascular diseases, are the leading causes of death worldwide and are associated with significant morbidity as well. The World Health Organization predicts around 23 million deaths due to cardiovascular diseases for the year 2030 [54]. Current state-of-the art treatment strategies for patients with occlusive diseases comprise percutaneous transluminal angioplasty (PTA), percutaneous transluminal coronary angioplasty (PTCA), or bypass surgery. However, certain patients do not require invasive procedures, because they benefit from the growth of pre-existing arteriolar collaterals into functional arteries, compensating for the loss of an artery due to occlusion (Figure 2). This process, defined as arteriogenesis [55,56], may be associated with patient survival. Indeed, a recent meta-analysis provided evidence that patients with high coronary collateralization showed a reduced risk of mortality [57]. Accordingly, much effort is made to decipher the molecular mechanisms of arteriogenesis aiming to identify drug targets and to enable clinicians to promote collateral-artery growth non-invasively.

### 3.1. Innate Immunity

Intensive investigations over the last 20 years revealed that arteriogenesis is mediated by a local and temporary inflammatory response. Upon the narrowing of an artery, e.g., due to atherosclerotic plaque formation, blood flow is re-directed into pre-existing arteriolar connections. These vessels now experience an increased mechanical load, i.e., shear stress, which activates the endothelium of the small arterioles, finally resulting in collateral artery growth [58]. This inflammatory process is initiated by platelets [59] and the subsequent steps appear to be a blueprint of the inflammatory cell responses, known for the innate immunity process. Upon transient interaction with the stressed endothelium, mediated by the platelet receptor glycoprotein 1bα (GPIbα), P-selectin is expressed on the surface of platelets [60]. Subsequently, P-selectin binds to P-selectin glycoprotein ligand 1 (PSGL-1) provoking platelet-neutrophil aggregate (PNA) formation, which is associated with the activation of neutrophil NADPH oxidase 2 (Nox2) to produce high doses of reactive oxygen radicals (ROS) [61]. In the perivascular space, these ROS activate mast cells to de-granulate, which, in turn, create an inflammatory environment. By increasing the bioavailability of tumor necrosis factor α (TNFα) and monocyte chemoattractant protein-1 (MCP-1), mast cells recruit neutrophils in a positive feedback loop and contribute to the recruitment of T cells and macrophages [61]. Although the functional role of T cells in arteriogenesis remains to be elucidated, macrophages play a crucial role for collateral artery growth by supplying growth factors and cytokines to the growing vessel [62].

### 3.2. Mechanosensing/Shear Stress

Mechanosensing plays an important role in all kind of physiological processes during embryonic development, e.g., in pruning of the immature vascular plexus [64], but also in the adult organism. Sensing of mechanical forces is not only involved in touching or hearing, but plays an important role in the cardiovascular system, in vascular homeostasis, atherosclerosis, and also arteriogenesis [58,65]. For laminar shear stress, which is the triggering force for arteriogenesis, manifold sensors (underlined in the text below) have been identified [66]. Interestingly, some of them are identical to receptors described for MK (see above), or induce identical signal transduction cascades, suggesting that MK is involved in the process of mechanosensing.

The luminal, polyanionic endothelial cell glycocalyx consists of different glycoproteins, hyaluronic acid, and proteoglycans such as syndecans. Due to its negative charge, this extended inner surface layer of all blood (and lymphatic) vessels allows the binding of a diverse range of positively charged proteins, with a variety of functions [66]. As the glycocalyx mitigates the variable shear forces experienced by endothelial cells at different locations in the vascular tree, cytoskeleton-linked syndecans (particularly syndecan-1) appear to be responsible for mechanical load transfer into cells where the translation into chemical messages occurs [67,68]. Syndecans not only act as co-receptors for cytokines, but also cooperate with integrins [67] and are involved in flow-induced NFκB activation [69]. Moreover, syndecan-1 has been demonstrated to play a role in Akt activation [70], being part of the phosphoinositide 3-kinase/Akt/endothelial nitric oxide synthase (PI3K/Akt/eNOS) pathway. However, mechanosensors such as primary cilia, and ion channels such as the transient receptor potential vanilloid 4 (TRPV-4), which are involved in endothelium calcium influx, were also related to nitric oxide (NO) production and accordingly to vasodilation [71,72]. 

Together with their associated seven transmembrane receptors, heterotrimeric G-proteins participate in shear stress-induced signaling by forming mechanosensitive complexes with other mechanosensors. For example, the G-protein Gαq/11 forms a complex with platelet-endothelial cell adhesion molecule-1 (PECAM-1), stabilized by heparan sulfates [73]. While PECAM-1 and integrins activate shear stress-evoked PI3K signaling essential for eNOS activation, a mechanosensory trimolecular complex consisting of PECAM-1, vascular endothelial cell cadherin (VE-Cadherin), and vascular endothelial growth factor receptor 2 (VEGFR2), has been shown to be involved in shear stress sensing relevant for collateral-artery growth [74].

### 3.3. Vascular Cell Proliferation

#### 3.3.1. Vascular Endothelial Growth Factor A (VEGF-A)

VEGF-A is well described to promote angiogenesis by interacting with VEGFR2. In contrast to arteriogenesis, which involves the proliferation of endothelial cells and smooth muscle cells, angiogenesis solely relies on the proliferation of endothelial cells forming capillaries. Capillaries have the function to distribute oxygen and metabolites locally, e.g., under conditions of tumor growth. However, in ischemic tissue, capillaries remove cell debris (Figure 2). Therefore, only collateral arteries, but not capillaries, can compensate for the loss of an artery caused by stenosis.

The functional role of VEGF-A in arteriogenesis has been controversially discussed for a long time, particularly based on the findings that administration of VEGF-A can hardly improve collateral-vessel formation. However, the blockade of VEGFR2 severely interfered with collateral-artery growth [75]. We were recently able to show that the bioavailability of VEGF-A is significantly increased after induction of arteriogenesis. Such elevated VEGF-A levels are critical and sufficient to promote collateral artery growth, whereas low concentrations of VEGF-A, as observed in MK-deficient mice, strongly hampered arteriogenesis. Moreover, treatment of wild-type mice with VEGF-A did not further promote the process of collateral artery growth [76]. Neither during the process of arteriogenesis, nor during the process of angiogenesis, VEGF-A is locally supplied by the tissue but by leukocytes, recruited to the sites of vessel growth [77,78]. For the process of angiogenesis, it has been demonstrated that chemokine (C-X-C motif) ligand 1 (CXCL-1), which is considered as the murine analogue to interleukin-8 (IL-8), and macrophage inflammatory protein-2 (MIP-2) locally recruit neutrophils, and that the release of VEGF-A from neutrophils is strictly dependent on CXCL-1/MIP-2 [78]. For arteriogenesis, it has been shown that CXCL-1 is upregulated in endothelial cells under conditions of increased shear stress in vivo [79] and in vitro [80]. Moreover, platelets, which we have shown to play an important role in arteriogenesis and to be involved in activating neutrophils by PNA formation, are a rich source of CXCL-1 [59,61,76]. In a recent study, it was shown that administration of CXCL-1 significantly promoted arteriogenesis, while blocking its receptor chemokine (C-X-C motif) receptor 2 (CXCR2) strongly interfered with collateral artery growth [79]. While CXCL-1 is relevant for initial neutrophil recruitment and release of VEGF-A in the early stages of arteriogenesis [61,76,78], MCP-1 likely overtakes these functions for macrophages [81,82], which become subsequently recruited to the perivascular tissue of growing collaterals as indicated before [61].

Interestingly, VEGF-A is only relevant for the proliferation of endothelial cells in growing collaterals, but not for smooth muscle cells [76]. This is even more astonishing as the activation of the VEGFR2/Neuropilin (NRP)1 receptor complex by VEGF-A [76,83] induces the release of von Willebrand factor (vWF) from endothelial cells [84]. vWF presents the major ligand of the platelet receptor GPIbα, and this interaction may initiate the inflammatory cascade, which is critical for the process of arteriogenesis.

#### 3.3.2. Midkine

Using a murine hindlimb model of collateral artery growth, we have recently shown that the process of arteriogenesis was severely compromised in MK-deficient mice, which was caused by a reduced bioavailability of VEGF-A [76]. MK deficiency resulted in hypertrophic outward remodeling, a process occurring when endothelial cell proliferation is reduced, while the proliferation of medial and adventitial cells remains unaffected [85]. MK was present in high amounts in neutrophils and macrophages [76,86], and indeed our results evidenced that leukocyte-derived MK was essential for collateral artery growth. A recent study demonstrated that overexpression of MK raised the expression level as well as the cellular release of VEGF-A [87]. Together, these data indicate that endothelial cell proliferation in arteriogenesis is dependent on the function of MK to mediate leukocyte (neutrophil and macrophage)-derived bioavailability of VEGF-A. We have previously shown that angiogenesis is severely impaired in MK-deficient mice, suggesting that MK also regulates the bioavailability of leukocyte-derived VEGF-A for endothelial cell proliferation in capillary sprouting [37]. These findings might have a major impact on the treatment of highly vascularized tumors as well, as tumor cells are likely to produce MK [5] to promote or even induce the vascularization of the tumor tissue itself. Accordingly, MK may represent a powerful tumor target. Further in-depth studies are required to prove this hypothesis. Whether there is a relationship between MK and the truncated variant of the somatostatin receptor subtype 5, sst5TMD4, which has been shown to elevate levels of VEGF when expressed in breast cancer cell lines [88], remains to be investigated.

Leukocyte recruitment and extravasation consists of several serial steps, starting with the capturing of free-flowing leukocytes, followed by leukocyte rolling on the endothelium, firm arrest, and finally diapedesis [89]. During rolling, leukocytes are activated by pro-inflammatory cytokines such as CXCL-1, which is associated with a rapid conformational change of β_2_ integrins [90,91,92]. The high-affinity conformation of β_2_ integrins is relevant for firm arrest of leukocytes to the endothelium by binding to ICAM-1 [93,94]. In a previous study, we have shown that MK supports adhesion of neutrophils by promoting the high-affinity conformational change of β_2_ integrins [27]. Another study demonstrated that activation of β_2_ integrins by MCP-1 resulted in increased expression levels of VEGF-A in macrophages [82].

**Figure 3:**
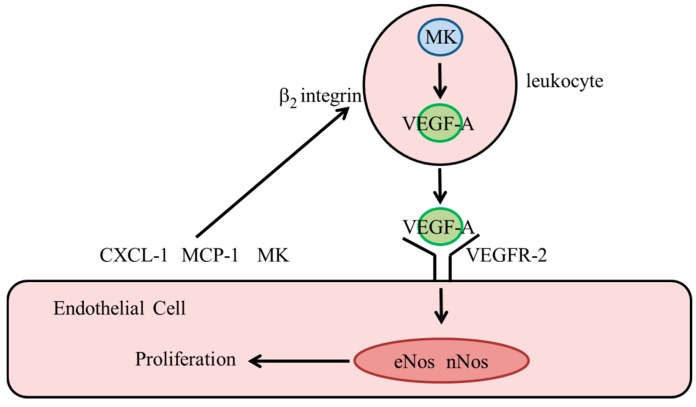
Proposed model for the mechanistic function of MK in regulating vascular endothelial cell proliferation in arteriogenesis. During recruitment, leukocytes get in close contact with the endothelial cell surface, which is covered with CXCL-1, MCP-1 and MK. Upon integrin β_2_ activation, VEGF-A is increased, expressed, and released from leukocytes. However, not only MK stored on endothelial cell surface, but also MK stored in leukocytes might contribute to increased bioavailability of VEGF-A. Upon binding to VEGFR2, VEGF-A promotes the expression of eNOS and nNOS relevant for endothelial cell proliferation. Adapted from Lautz et al., 2018 [76].

In terms of arteriogenesis, the following scenario is proposed (Figure 3): During the recruitment process, leukocytes are in close contact with the inflamed endothelial surface, to which MCP-1, CXCL-1, and possibly MK are bound via ionic interactions. Juxtracrine signaling of these immobilized cytokines results in the stimulation of approximated leukocytes with the inside-out activation of their β_2_ integrins as well as an increased expression level and release of VEGF-A. Since bone marrow cell-derived, but not endothelial MK appears to be critical for arteriogenesis, MK concentrated within the glycocalyx of endothelial cells might be derived from bone-marrow cells. Moreover, MK expressed and stored in leukocytes might also contribute to the increased bioavailability of VEGF-A. Further research needs to uncover the cellular sources of MK and its signal-transduction mechanism in this particular context. 

#### 3.3.3. Nitric Oxide Synthases

All isoforms of nitric oxide synthases, i.e., eNOS, neuronal NOS (nNOS), and inducible NOS (iNOS) have been described to contribute to arteriogenesis [76,95,96]. For angiogenesis, NO derived from eNOS has been implicated in endothelial cell proliferation [97,98,99]. As reduced perfusion recovery of eNOS-deficient mice was improved by administration of an NO donor, it was originally hypothesized that eNOS in arteriogenesis is only relevant for vasodilation [96,100]. We have recently shown that nNOS deficiency was not associated with reduced perfusion recovery in a hindlimb model of arteriogenesis. However, in contrast to eNOS deficiency, administration of an NO donor showed deleterious effects in nNOS-deficient mice [76]. Interestingly, MK-deficient mice showed reduced expression levels of eNos and nNos. Accordingly, we were interested whether the reduced levels of NO synthases were causative for impaired arteriogenesis in MK-deficient mice. Our results evidenced that treatment with an NO donor completely rescued diminished endothelial cell proliferation and hence the process of arteriogenesis in MK-deficient mice [76].

While eNOS has mainly been described to produce NO, nNOS is more involved in the generation of H_2_O_2_ [101,102], although uncoupling of eNOS promotes H_2_O_2_ production as well [103]. Both NO [97] as well as H_2_O_2_ [104,105] have been demonstrated to contribute to endothelial cell proliferation, and both NOS isoforms, eNOS and nNOS, can replace each other [106]. In a rodent model of cerebral aneurysm formation, the pathology outcome was neither affected in eNOS- nor in nNOS-deficient mice, but severely increased in mice deficient for both genes. While neither eNOS- nor nNOS-deficiency affects endothelial cell proliferation during arteriogenesis, it is strongly compromised in MK-deficient mice. Owing to the reduced expression levels of both eNOS and nNOS in MK-deficient mice, it is fair to deduce that both NOS isoforms can substitute for each other in their functional activities during arteriogenesis in terms of promoting endothelial cell proliferation.

It has been described that NO and VEGF-A affect their expression in a bi-directional manner [107,108]. In MK-deficient mice, administration of an NO donor did not restore the reduced VEGF-A level, whereas administration of VEGF-A rescued the reduced expression of eNOS and nNOS [76]. These data clearly demonstrate that during arteriogenesis, VEGF-A apparently regulates the expression levels of both NOS that are crucial for vascular endothelial-cell proliferation. The fact that the upstream MK regulates the bioavailability of VEGF-A renders MK a conductor to orchestrate endothelial cell proliferation during arteriogenesis (Figure 3).

### 3.4. Vasodilation

NO is the most potent known vasodilator. We have recently shown that administration of VEGF-A did not promote vasodilation in wild-type mice, whereas administration of MK significantly increased vasodilation [76]. These data indicate that MK activates NOS independently from the VEGF-A pathway. It implies a direct action of MK on receptors of endothelial cells. It has previously been demonstrated in human umbilical vein endothelial cells (HUVECs) that stimulation with MK resulted in binding and phosphorylation of the MK receptor ALK and activation of PI3K and MAPK signaling [25]. In a murine model of myocardial infarction, it was shown that enhanced angiogenesis, induced by exogenous administered MK, was associated with PI3K/Akt and MAPK activation, and expression of syndecan-1, -3, and -4 [109]. Syndecan-1 is well-described to promote Akt activation [70]. Moreover, syndecans are co-receptors of integrins, and both, syndecan-1 as well as integrins, have not only been described to be receptors for MK and to be involved in Akt activation, but also to be involved in mechanosensing (see above).

There is a variety of studies relating PI3K to nNOS expression [110], as well as nNOS [111] and eNOS [112] activation. Interestingly, retinoic acid, which is described to induce the expression of MK [7], has also been shown to induce nNOS expression via the PI3K/Akt pathway [113]. Together, the available data suggest that a receptor complex on endothelial cells, which is involved in mechanosensing, is responsible for MK-mediated signal transduction pathways, resulting in NOS activation, which in turn is responsible for vasodilation. However, it appears that also intracellular MK can activate PI3K signaling and hence vasodilation [114].

Fujiwara et al. have recently described that MK is likely to act on growth hormone cells via the protein tyrosine phosphatase receptor-type Z, Ptprz1 [115], and Rubinek and Modan-Moses suggested that klotho is a direct regulator of growth hormone secretion [116,117]. In the literature, there are a several reports available demonstrating that growth hormone promotes vasodilation and restores endothelial function [118,119,120]. Accordingly, it is tempting to speculate that midkine might also promote vasodilation indirectly by increasing the bioavailability of growth hormone. However, further studies are necessary to confirm the relation between MK and growth hormone.

**Figure 4:**
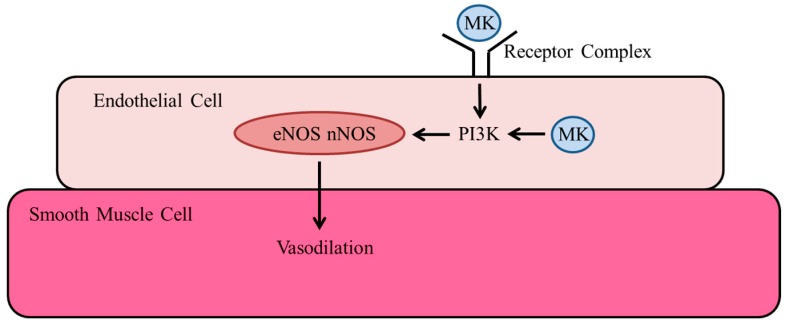
Proposed model for the function of MK to promote vasodilation. Upon binding to a receptor complex, MK activates NOS via the phosphoinositide 3-kinase (PI3K)/Akt pathway. However, MK located in endothelial cells might also contribute by directly activating this signal transduction cascade. Adapted from Lautz et al., 2018 [76].

In one of our recent studies, we have shown that administration of MK to wild-type mice resulted in prolonged vasodilation, an effect that was not observed when mice were treated with an NO donor [76]. In patients, currently available NO donors do not show long-term vasodilation due to rapid tolerance, and here, MK may provide a novel alternative to induce the activation of NOS (Figure 4).

## 4. Conclusions

MK was originally identified as a modulator of embryonic development and was later assigned a role in tumor growth and inflammatory diseases. Meanwhile, MK is discovered as major determinant of the cardiovascular system involved in such important processes as translation of mechanical forces. It is not only engaged in vascular cell proliferation but even in regulation of the circulation.

## Figures and Tables

**Table 1:** Midkine receptors.

Receptor Family	Receptor	Reference
Protein tyrosine phosphatase (PTP)	PTPζ	Maeda et al., 1999 [23]
		Qi et al., 2001 [24]
LDL-receptor-related protein (LRP)	LRP-1	Muramatsu et al., 2000 [26]
	megalin/brushin	Muramatsu et al., 2000 [26]
	LRP-6	Muramatsu et al., 2004 [28]
	apoE receptor-2	Muramatsu et al., 2000 [26]
Integrins	α_4_β_1_	Muramatsu et al., 2004 [28]
	α_6_β_1_	Muramatsu et al., 2004 [28]
Notch	notch2	Huang et al., 2008 [35]
Receptor tyrosine kinase	ALK	Stoica et al., 2007 [25]
Glycosaminoglycans	Heparan sulfate trisulfated units	Ueoka et al., 2000 [29]
	Chondroitin sulfate E units	Ueoka et al., 2000 [29]
	Syndecan-1	Nakanishi et al., 1997 [32]
	Syndecan-3	Nakanishi et al., 1997 [32]
	Glypican-2	Kurosawa et al., 2001 [31]

Table adapted from Weckbach et al. [6].

## Abbreviations

MK	midkine
PTN	pleiotrophin
PTP*ζ*	receptor-like protein tyrosine phosphatase β/protein tyrosine phosphatase *ζ*
WT-1	Wilms tumor suppressor gene
PI3K	phosphatidylinositol 3-kinase
MAPK	mitogen-activated protein kinase
ALK	anaplastic lymphoma kinase
LRP	low-density-lipoprotein receptor-related protein
ICAM-1	intercellular adhesion molecule-1
NFκB	nuclear factor kappa-light-chain-enhancer of activated B cells
EAE	autoimmune encephalomyelitis
CR	cysteine rich complement-like repeats
PTA	percutaneous transluminal angioplasty
PTCA	percutaneous transluminal coronary angioplasty
GPIbα	glycoprotein 1bα
PSGL-1	P-selectin glycoprotein ligand 1
PNA	platelet-neutrophil aggregate
Nox2	neutrophil-NADPH oxidase 2
uPA	urokinase plasminogen activator
ROS	reactive oxygen species
TNFα	tumor necrosis factor α
MCP-1	monocyte chemoattractant protein-1
LAD	Left Anterior Descending
LCX	Left Circumflex
NO	nitric oxide
PECAM-1	platelet adhesion molecule-1
VE-Cadherin	vascular endothelial cell cadherin
VEGFR2	vascular endothelial growth factor receptor 2
TRPV-4	transient receptor potential vanilloid 4
VEGF-A	vascular endothelial growth factor A
CXCL-1	chemokine (C-X-C motif) ligand 1
MIP-2	macrophage inflammatory protein-2
CXCR2	chemokine (C-X-C motif) receptor 2
IL-8	CXCL-8/interleukin-8
NRP1	Neuropilin 1
vWF	von Willebrand factor
Mac-1	macrophage-1 antigen
LFA-1	lymphocyte function-associated antigen 1
iNOS	inducible NOS
nNOS	neuronal NOS
CA	cerebral aneurisma
